# The effectiveness of a primary care-based collaborative care model to improve quality of life in people with severe mental illness: PARTNERS2 cluster randomised controlled trial

**DOI:** 10.1192/bjp.2023.28

**Published:** 2023-06

**Authors:** Richard Byng, Siobhan Creanor, Benjamin Jones, Joanne Hosking, Humera Plappert, Sheriden Bevan, Nicky Britten, Michael Clark, Linda Davies, Julia Frost, Linda Gask, Bliss Gibbons, John Gibson, Pollyanna Hardy, Charley Hobson-Merrett, Peter Huxley, Alison Jeffery, Steven Marwaha, Tim Rawcliffe, Siobhan Reilly, Debra Richards, Ruth Sayers, Lynsey Williams, Vanessa Pinfold, Maximillian Birchwood

**Affiliations:** Community and Primary Care Research Group, University of Plymouth, UK; Department of Health and Community Sciences, University of Exeter, UK; Institute for Mental Health, University of Birmingham, UK; Birmingham Clinical Trials Unit, University of Birmingham, UK; Care Policy and Evaluation Centre, London School of Economics and Political Science, UK; Division of Population Health, University of Manchester, UK; Institute for Mental Health, Coventry and Warwickshire Partnership NHS Trust, UK; The McPin Foundation, UK; National Perinatal Epidemiology Unit, Oxford Population Health, Nuffield Department of Population Health, University of Oxford, UK; School of Medical and Health Sciences, University of Bangor, UK; Institute for Mental Health, University of Birmingham, UK; and Institute for Mental Health, Birmingham and Solihull Mental Health NHS Foundation Trust, UK; Lancashire Care NHS Trust, UK; Centre for Applied Dementia Studies, Faculty of Health Studies, University of Bradford, UK; Health Sciences, University of Warwick, UK

**Keywords:** Primary care, randomised controlled trial, schizophrenia, bipolar affective disorders, outcome studies

## Abstract

**Background:**

Individuals living with severe mental illness can have significant emotional, physical and social challenges. Collaborative care combines clinical and organisational components.

**Aims:**

We tested whether a primary care-based collaborative care model (PARTNERS) would improve quality of life for people with diagnoses of schizophrenia, bipolar disorder or other psychoses, compared with usual care.

**Method:**

We conducted a general practice-based, cluster randomised controlled superiority trial. Practices were recruited from four English regions and allocated (1:1) to intervention or control. Individuals receiving limited input in secondary care or who were under primary care only were eligible. The 12-month PARTNERS intervention incorporated person-centred coaching support and liaison work. The primary outcome was quality of life as measured by the Manchester Short Assessment of Quality of Life (MANSA).

**Results:**

We allocated 39 general practices, with 198 participants, to the PARTNERS intervention (20 practices, 116 participants) or control (19 practices, 82 participants). Primary outcome data were available for 99 (85.3%) intervention and 71 (86.6%) control participants. Mean change in overall MANSA score did not differ between the groups (intervention: 0.25, s.d. 0.73; control: 0.21, s.d. 0.86; estimated fully adjusted between-group difference 0.03, 95% CI −0.25 to 0.31; *P* = 0.819). Acute mental health episodes (safety outcome) included three crises in the intervention group and four in the control group.

**Conclusions:**

There was no evidence of a difference in quality of life, as measured with the MANSA, between those receiving the PARTNERS intervention and usual care. Shifting care to primary care was not associated with increased adverse outcomes.

## Clinical need

Individuals diagnosed with severe mental illness (SMI) represent more than 1% of the population in the UK,^1–3^ and can have significant psychological, physical and social problems. Yet an estimated 50–70% receive no specialist mental healthcare and only limited support from general practice.^4^ Quality of life is often poor, as health problems can affect education and employment, difficulties with relationships and low confidence, with recovery an idiosyncratic process.^5^ People with a schizophrenia or bipolar diagnosis have a significantly reduced life expectancy compared with the general population.^6^ Two-thirds of the mortality gap can be explained by physical disorders, predominantly associated with cardiovascular problems and diabetes,^7^ and there are clear health inequalities faced by people with SMI.^8^

## Policy context

To counter these problems, policy in England promotes joined-up emotional, social, psychological and physical care, including integrating primary and secondary care.^9^ The recent National Health Service (NHS) community mental health transformation policy for England^10^ aims to ensure those with psychosis who need care are supported, and for much of this to be carried out as a collaboration between specialist providers, primary care and third-sector organisations. The wider comprehensive model for personalised care emphasises shared decision-making, coaching, social prescribing and having a single plan of care with one key coordinating practitioner.^11^ Such person-centred approaches are seen as fundamental to the care delivered by primary care networks, the main organisational unit for general practice-led, community-based healthcare in England.

## Current evidence

For individuals with SMI diagnoses there has been a significant focus on research addressing medication effectiveness, and psychosocial support for early intervention, whereas individuals with less intense, but nevertheless longstanding and substantial needs, have received less attention. Consequently, there is a relative lack of evidence to inform the policy ambitions of integrated person-centred care. Exceptions include the adaptation of collaborative care, developed for those with depression, to the needs of individuals with psychosis.^12^ Collaborative care normally includes a structured plan and enhanced approach to joint working between primary and secondary care, proactive review and supervision.^13^

## The PARTNERS2 programme

The PARTNERS2 programme is a primary care-based collaborative care model aimed to develop and evaluate collaborative care to address mental and physical care deficits for people with SMI already discharged from, or receiving low levels of, specialist care. Initial phases of the programme examined current care pathways, addressed trial science challenges and co-developed the intervention incorporating a coaching approach to ensuring focus of care is centred around the wishes of the individual, with additional motivational components to tackle physical health needs.^14,15^ Patient and public involvement was embedded throughout the whole programme and was key to development of the intervention.^16^ This paper describes the cluster randomised controlled trial designed to test the hypothesis that PARTNERS, a coaching-based model of collaborative care, would improve quality of life for people with SMI when compared with care as usual.

## Method

### Study design

We conducted this unmasked, general practice-based, cluster randomised, controlled open-label superiority trial across four regions in England, UK, with a cluster defined as a general practice.

The trial was delivered according to the protocol,^17^ with amendments made to continue trial delivery and follow-up after the start of the COVID-19 pandemic in early 2020. The authors assert that all procedures contributing to this work comply with the ethical standards of the relevant national and institutional committees on human experimentation and with the Helsinki Declaration of 1975, as revised in 2008. All procedures involving human patients were approved by the West Midlands-Edgbaston Research Ethics Committee (reference number 14/WM/0052). Local NHS approvals were obtained before the start of recruitment in each region from 27 February 2018. We had an independent Data Monitoring Committee and Trial Steering Committee, as agreed with the funder. The trial is registered with the ISRCTN Registry (identifier ISRCTN95702682).

### Participants

General practices in England were eligible to participate if they were providing care in the participating areas (Birmingham/Solihull, Plymouth, Cornwall, Somerset); had ≥16 patients on their Quality and Outcomes Framework (QOF) register for SMI; and were willing and able to host the intervention. We initially recruited practices in Lancashire, but had to discontinue following changes in services there.

We included people aged ≥18 years registered with participating general practices; diagnosed with schizophrenia, bipolar or other type of psychosis diagnosis; and with evidence of care needs for this diagnosis in the past 2 years, but not currently requiring acute or ongoing secondary multidisciplinary mental healthcare. We aimed for an average of six individuals per practice. We excluded people who could not give informed consent, who needed access to translation services, for whom the general practitioner (GP) believed that it was not in their best interests, who were currently participating in a trial for psychosis, who were receiving secondary care for dementia or intellectual disability, and/or individuals with significant substance or alcohol issues.

Potential participants using secondary care were identified from secondary care records and other potential participants ‘seen only in primary care’ from general practice records. Information packs were posted to potential participants, inviting participation and emphasising how patient researchers were part of the team.^16,18^ Those who did not respond were invited to an appointment or could opt out, and were telephoned where possible. Researchers met those interested in participating and obtained written informed consent.

### Randomisation and masking

Once all participants in a practice were recruited and baseline data were collected, we assigned the practice to the PARTNERS intervention or care as usual (control) at a 1:1 allocation, using a computer-generated minimisation algorithm, with allocation minimised on geographical area and estimated practice psychosis population size, according to the QOF register. Allocation was undertaken by a Clinical Trials Unit researcher not involved in delivery or analysis of the trial, and communicated to the practice and participants by letter or preferred method of contact.

It was not possible to blind staff in the general practice or the participants because of the nature of the psychosocial intervention. Researchers conducting follow-up assessments were not blinded for logistical reasons, as they were responsible for maintaining relationships with intervention practitioners and general practices. Statisticians were masked to allocation during primary analyses.

### Intervention and usual care descriptions

The PARTNERS intervention was developed in line with the Medical Research Council's complex intervention guidance.^14,15,19^ The intervention aims to enable contact and ongoing continuity with a mental health worker (a ‘care partner’) based in primary care, who delivers a coaching approach and liaises with primary care and other services. The theoretical basis for benefit is that one or more of multiple diverse mechanisms to improve health or well-being can be triggered and contribute to improved quality of life. The manualised model (available on request) aims to improve emotional, social, mental and physical health outcomes for people with psychosis. Care partners work with participants to develop a shared understanding about current problems and goals, and utilise coaching and motivational techniques with the intention of encouraging participants to be more confident and proactive about their health through developing self-management skills, and achieve personal goals related to their health or other aspects of their lives. Care partners work collaboratively with primary care, secondary care and other organisations. Place and method of contact (face to face, telephone, text) is flexible, starting with fortnightly contact, stepping down to every 3 months for some if agreed, but stepping up, potentially including referral into secondary care, if required.

In the trial, participants received the PARTNERS intervention for up to 12 months, including a 2-month transition period back to usual care. Participants in the intervention group and already under secondary care either had this care paused or the intensity was reduced according to a protocol specific to each NHS provider. Care partners came from a variety of backgrounds, including occupational therapists, social workers, nurses and support workers. They received 2–3 days of initial training, top-up training throughout the study, and supervision. Supervisors included mental health nurses and psychiatrists who received training in the PARTNERS model. The co-chief investigator (R.B.), an accredited GP with a special interest in mental health, provided cover for supervision during periods of illness or absence. Participants allocated to the control arm continued to receive usual care only, either within primary care only or also within secondary care.

Fidelity of the delivery of intervention against the theoretical model was assessed through a comprehensive mixed-methods process evaluation. The proportion of time care partners were in post and numbers of sessions each individual attended with a care partner are reported in this paper.

### Outcomes

Participant outcome data were collected at or shortly after consent and at a single follow-up point. Follow-up was originally planned to be 10 months (±1 month) after randomisation of the practice. The anticipated operational problems of following up the high number of individuals recruited in the final month (*n* = 56) led to a decision to shorten the follow-up period to 9 months (±1 month) for participants recruited at the end of the recruitment window (February 2020). This ensured follow-up completion by December 2020, keeping within the timelines for the research funding.

The primary outcome measure was quality of life as measured by change in score on the participant-reported Manchester Short Assessment of Quality of Life (MANSA) version 2,^20^ from baseline to follow-up. This self-complete questionnaire comprises objective and subjective questions across different life domains (work and education, personal finances, leisure activities, social life, living situation, family life, personal safety and health). It was chosen through a consensus stakeholder process because it has shown sensitivity to change in this population, is short and captures important outcomes that the intervention was designed to achieve.

Participant-reported secondary outcomes collected at baseline and follow-up were the Time Use Survey (TUS; assessing time in structured activity),^21^ Questionnaire about the Process of Recovery (QPR-15),^22^ full and short versions of the Warwick-Edinburgh Mental Wellbeing Scale ((S)WEMWBS),^23^ Brief INSPIRE (a measure of experience of care),^24^ ICEpop CAPability (ICECAP-A; to measure social well-being)^25^ and the five-level EuroQol five-dimensional (EQ-5D-5L; for health status).^26^

Data about mental healthcare service use were collected from secondary care records. Safety outcomes, analysed from the date of practice unmasking (randomisation) through to 10 months after randomisation, were the number of psychiatric hospital admissions, number of in-patient days as a result of psychiatric admission, number of episodes under home treatment (crisis care) and total days under home treatment (crisis care). Details of serious adverse events (e.g. hospital admissions) were also collected after identification during follow-up by researchers and/or practitioners during routine or PARTNERS care.

### Statistical analysis

The original recruitment target was 336 participants across approximately 56 general practice clusters, to detect a mean between group difference of 0.45 points^27^ in the overall MANSA score, with 90% power and two-sided 5% significance level, assuming an s.d. of 0.9, mean of six participants recruited per cluster (general practice), coefficient of variation of cluster size of 0.74, intracluster correlation (ICC) of 0.05, 20% drop-out at the individual participant level and 10% drop-out at the cluster level. The target standardised effect size was therefore 0.5, selected in line with the target effect size in the DIALOG+ trial.^27^ The protocol included an interim blinded review of the sample size assumptions, including exploring adjusting for the correlation between baseline and follow-up MANSA scores. From the first 39 participants the observed correlation was 0.69 (80% CI 0.56–0.79); we conservatively allowed for a correlation of 0.5 in a revised sample size calculation. Retaining the other underpinning assumptions of the original calculation indicated requiring primary outcome data from 180 participants (recruitment target of 270 participants allowing for drop-out) or 140 participants (recruitment target of 204 participants) to achieve 90% or 80% power, respectively. To adhere to funder-mandated timescales, the revised aim (agreed with the oversight committees) was to recruit 204 participants from approximately 34 general practices.

A detailed statistical analysis plan was approved before locking the trial database.^28^ All primary analyses followed the modified intention-to-treat principle (i.e. analysis of the complete-case data according to allocated group). Two prespecified sensitivity analyses of the primary outcome were on a per-protocol basis. Analyses of safety outcomes were on the as-treated basis: for safety event/episode analysis, intervention participants were categorised as being ‘treated’ if they had had at least one interaction with their care partner before the date of onset of the episode/serious adverse event. Analyses were undertaken in Stata version 16.0 for Windows (StataCorp LLC, College Station, Texas, USA; https://www.stata.com/) and R version 4.0.3 for Windows (R Core Team, R Foundation for Statistical Computing, Vienna, Austria; https://cran.r-project.org/bin/windows/base/).

The change between baseline and follow-up in the primary outcome of overall MANSA score was analysed with a random effects linear regression model, with cluster-level minimisation factors (region and practice size) and individual-level baseline overall MANSA score included as fixed effects covariates, and general practice as a random effect. Prespecified sensitivity analyses of the primary outcome assessed the robustness of the primary analysis: (a) per-protocol analysis of participants with follow-up data within their prespecified window; (b) per-protocol analysis to assess the impact of availability of care partner to deliver the intervention, excluding practices where a care partner was available <70% of time; (c) using multiple imputation to assess the effect of missing primary outcome data, including the number of care partner interactions as an auxiliary variable; and (d) complier-average causal effect analyses using two prespecified definitions of fidelity (attendance at a minimum of six care partner sessions, either face to face, by telephone or virtually, with each session lasting a minimum of 10 min; and attendance at a minimum of four care partner sessions during which goals were discussed).

To explore the effects of the COVID-19 pandemic, participants were categorised according to whether their follow-up data was collected before or during the pandemic, with three additional prespecified sensitivity analyses for both the primary outcome and the secondary outcome TUS: (a) separate models for participants providing follow-up data before and after the start of the first UK lockdown, (b) adding an interaction term of allocated group/COVID-19-affected categorisation to the primary analysis model and (c) adding the COVID-19-affected categorisation covariate as an adjustment to the primary analysis model. We also undertook four planned subgroup analyses (region, practice size (small versus large), diagnostic group (bipolar disorder versus schizophrenia or other psychosis) and usual care provider at screening (primary versus secondary care)) of the primary outcome by including the interaction effect of allocated group and the subgroup in separate regression models. All fully adjusted sensitivity and subgroup analyses included the minimisation factors, general practice as a random effect and controlled for the corresponding baseline score.

We analysed continuous secondary outcomes with similar random effects linear regression modelling. Two identified outliers were removed from the analyses of the TUS. We were unable to complete the planned analysis of the number of healthcare monitoring outcomes because of limited availability of data (primary care notes reviews were carried out for 32 participants). After discussion with the oversight committees, no inferential analyses were undertaken of the Brief INSPIRE because of both the levels and patterns of missing data at both baseline and follow-up. No inferential analyses were planned/undertaken of the lifestyle outcomes.

## Results

**Fig. 1:**
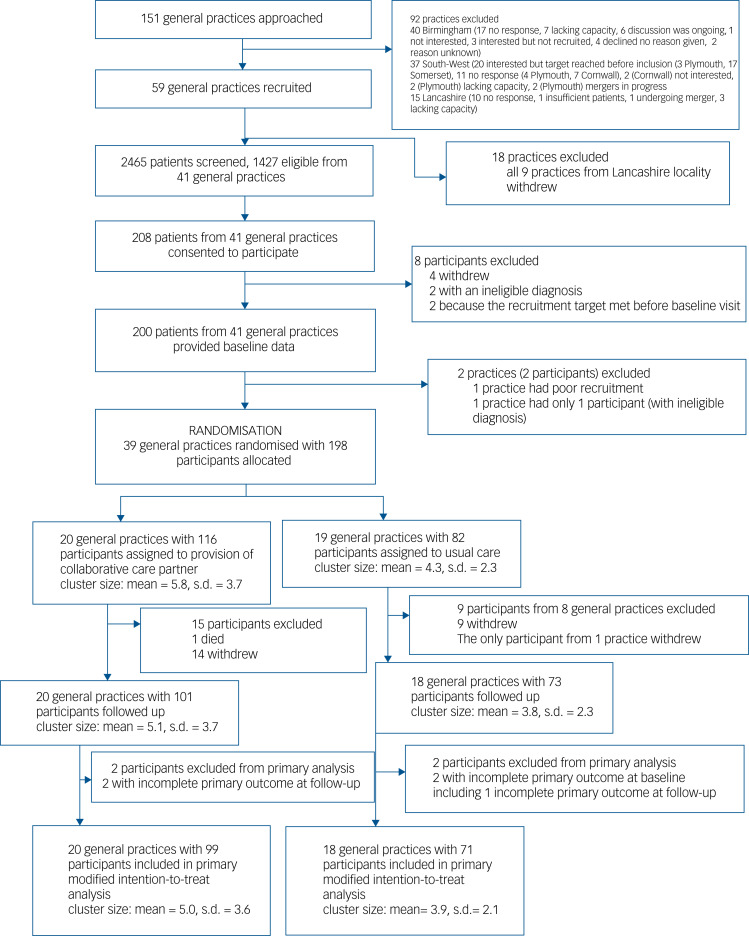
Consolidated Standards of Reporting Trials (CONSORT) diagram.

We recruited general practices from January 2018 to January 2020, and participants between June 2018 and February 2020. We randomly assigned 39 general practices to either the PARTNERS2 intervention group (20 practices, 116 participants) or control group (19 practices, 82 participants) (Fig. 1).

The two allocated groups were broadly similar in terms of most baseline characteristics of participants (Table 1). The mean age was 53 years and 176 (89%) were White. Over half (58%) of participants had a record of bipolar disorder and roughly a fifth (22%) had a record of schizophrenia or schizoaffective disorder. Overall, two-thirds of participants had their mental health needs cared for by primary care only, and a higher proportion of the intervention group were under primary care only, compared with the control group (72% *v*. 57%). Over 60% of participants were female and around 40% were single. Around 40% were current smokers. Under 10% had a formal carer, just over 40% had an informal carer and nearly half reported having some caring responsibilities themselves.

**Table 1:** Cluster and participant characteristics at baseline

	Intervention (20 practices; *n* = 116)	Control (19 practices; *n* = 82)	Overall (39 practices; *N* = 198)
Cluster characteristics			
Locality			
Birmingham	8/20 (40.0%)	8/19 (42.1%)	16/39 (41.0%)
Plymouth	5/20 (25.0%)	5/19 (26.3%)	10/39 (25.6%)
Cornwall	5/20 (25.0%)	5/19 (26.3%)	10/39 (25.6%)
Somerset	2/20 (10.0%)	1/19 (5.3%)	3/39 (7.7%)
Practice size			
Small	8/20 (40.0%)	7/19 (36.8%)	15/39 (38.5%)
Large	12/20 (60.0%)	12/19 (63.2%)	24/39 (61.5%)
Rurality			
Urban	15/20 (75.0%)	15/19 (78.9%)	30/39 (76.9%)
Rural	5/20 (25.0%)	4/19 (21.1%)	9/39 (23.1%)
Deprivation decile			
High (1–3)	12/20 (60.0%)	14/19 (73.7%)	26/39 (66.7%)
Medium (4–6)	5/20 (25.0%)	5/19 (26.3%)	10/39 (25.6%)
Low (7–10)	3/20 (15.0%)	0 (0%)	3/39 (7.7%)
Participant characteristics			
Age, years	53.1 (13.6) [25.2–78.3]	52.9 (12.1) [22.5–84.2]	53.0 (13.0) [22.5–84.2]
Gender			
Female	69/115 (60.0%)	55/82 (67.1%)	124/197 (62.9%)
Male	46/115 (40.0%)	27/82 (32.9%)	73/197 (37.1%)
Ethnicity			
White	105/115 (91.3%)	71/82 (86.6%)	176/197 (89.3%)
Mixed	4/115 (3.5%)	0 (0.0%)	4/197 (2.0%)
Asian	2/115 (1.7%)	3/82 (3.7%)	5/197 (2.5%)
Black	2/115 (1.7%)	6/82 (7.3%)	8/197 (4.1%)
Other	0 (0.0%)	1/82 (1.2%)	1/197 (0.5%)
Not known/not provided	2/115 (1.7%)	1/82 (1.2%)	3/197 (1.5%)
Relationship status			
Single	44/115 (38.3%)	34/82 (41.5%)	78/197 (39.6%)
Married	37/115 (32.2%)	18/82 (22.0%)	55/197 (27.9%)
Partner	16/115 (13.9%)	8/82 (9.8%)	24/197 (12.2%)
Divorced/separated	12/115 (10.4%)	19/82 (23.2%)	31/197 (15.7%)
Widowed	5/115 (4.3%)	3/82 (3.7%)	8/197 (4.1%)
Other	1 (0.9%)	0 (0.0%)	1/197 (0.5%)
Education			
Level 1–2 (e.g. GCSE/BTEC)	29/116 (25.0%)	23/82 (28.0%)	52/198 (26.3%)
Level 3–5 (e.g. A level, HND)	46/116 (39.7%)	27/82 (32.9%)	73/198 (36.9%)
Level 6–8 (e.g. degree, PhD)	27/116 (23.3%)	14/82 (17.1%)	41/198 (20.7%)
None	12/116 (10.3%)	17/82 (20.7%)	29/198 (14.6%)
Other	2/116 (1.7%)	1/82 (1.2%)	3/198 (1.5%)
Physical health conditions			
Chronic heart disease	10/116 (8.6%)	1/82 (1.2%)	11/198 (5.6%)
Cancer	4/116 (3.4%)	1/82 (1.2%)	5/198 (2.5%)
Stroke	6/116 (5.2%)	2/82 (2.4%)	8/198 (4.0%)
Bronchitis/stroke/emphysema	18/116 (15.5%)	7/82 (8.5%)	25/198 (12.6%)
Asthma	25/116 (21.6%)	18/82 (22.0%)	43/198 (21.7%)
Diabetes	14/116 (12.1%)	11/82 (13.4%)	25/198 (12.6%)
Epilepsy, seizures or fits	6/116 (5.2%)	6/82 (7.3%)	12/198 (6.1%)
Hypertension	29/116 (25.0%)	17/82 (20.7%)	46/198 (23.2%)
Liver disease	3/116 (2.6%)	8/82 (9.8%)	11/198 (5.6%)
Kidney disease	6/116 (5.2%)	7/82 (8.5%)	13/198 (6.6%)
Health check in the past 12 months			
No	36/114 (31.6%)	29/81 (35.8%)	65/195 (33.3%)
Yes	76/114 (66.7%)	49/81 (60.5%)	125/195 (64.1%)
Do not know	2/114 (1.8%)	3/81 (3.7%)	5/195 (2.6%)
Smoking			
Current smoker	51/116 (44.0%)	31/82 (37.8%)	82/198 (41.4%)
Cigarettes	11/51 (88.2%)	26/31 (83.9%)	71/82 (86.6%)
Pipe	0 (0.0%)	1/31 (3.2%)	1/82 (1.2%)
E-cigarette	11/51 (21.6%)	9/31 (29.0%)	20/82 (24.4%)
Times smoking per day	*n* = 48 17.7 (12.3) [1–60]	*n* = 28 17.1 (12.1) [2–60]	*n* = 76 17⋅5 (12.2) [1–60]
Alcohol			
Drinks alcohol	55/116 (47.4%)	36/82 (43.9%)	91/198 (46.0%)
Times drinking per week	*n* = 45 2.9 (2.2) [0–7]	*n* = 30 2.7 (2.3) [0–7]	*n* = 75 2.8 (2.2) [0–7]
Carer			
Has a formal carer	13/116 (11.2%)	5/82 (6.1%)	18/198 (9.1%)
Has an informal carer	46/116 (39.7%)	37/82 (45.1%)	83/198 (41.9%)
Childcare			
Had childcare responsibilities in the past month	27/116 (23.3%)	19/82 (23.2%)	46/198 (23.2%)
Hours of childcare in the past month	*n* = 23 Median: 75 IQR: [24–312]	*n* = 16 Median: 60 IQR: [22.5–269]	*n* = 39 Median: 64 IQR: [24–288]
Other caring responsibilities			
Has other caring responsibilities in the past month	20/116 (17.2%)	19/82 (23.2%)	39/116 (19.7%)
Hours of other caring responsibilities in the past week	*n* = 18 Median: 24 IQR: [8–60]	*n* = 17 Median: 32 IQR: [15–150]	*n* = 35 Median: 28 IQR: [10–90]
Household tasks			
Responsible for household tasks	106/115 (92.2%)	78/82 (95.1%)	184/198 (93.4%)
Hours of household tasks per week	*n* = 104 Median: 7 IQR: [4–14]	*n* = 75 Median: 7 IQR: [4–14]	*n* = 179 Median: 7 IQR: [4–14]
Mental healthcare provider (at baseline)			
Primary care	84/116 (72.4%)	47/82 (57.3%)	131/198 (66.2%)
Secondary care	32/116 (27.6%)	35/82 (42.7%)	67/198 (33.8%)
Mental health diagnosis			
Bipolar disorder	69/114 (60.5%)	44/80 (55.0%)	113/194 (58.2%)
Schizophrenia	24/114 (21.1%)	19/80 (23.8%)	43/194 (22.2%)
Other psychoses	21/114 (18.4%)	17/80 (21.3%)	38/194 (19.6%)
Age at mental health diagnosis	*n* = 102 35.3 (12.8) [14–70]	*n* = 77 34.8 (12.9) [12–66]	*n* = 179 35⋅1 (12.8) [12–70]
Mental health medication			
Antipsychotics	75/116 (64.7%)	61/82 (74.4%)	136/198 (68.7%)
Antidepressants	74/116 (63.8%)	47/82 (57.3%)	121/198 (61.1%)
Sedatives/hypnotics	28/116 (24.1%)	23/82 (28.0%)	51/198 (25.8%)
Mood stabilisers	62/116 (53.4%)	35/82 (42.7%)	97/198 (49.0%)
Other	9/116 (7.8%)	8/82 (9.8%)	17/198 (8.6%)

Data are displayed as *n*/*N* (%), mean (s.d.) and [range], or median (lower quartile, upper quartile) and [interquartile range (IQR)].

A care partner was in place for at least 70% of the intervention period for 75% (15 out of 20) of the intervention practices, and the majority of intervention group participants (91%) had at least one care partner interaction of any type. The level of participant engagement with the intervention is summarised in Supplementary Table 1 available at https://doi.org/10.1192/bjp.2023.28.

None of the general practices withdrew from the trial after randomisation, although one practice that only recruited one participant was not included in the comparative analyses because the participant withdrew before follow-up. Primary analysis of the primary outcome included 99 (85.3%) of the 116 randomised participants in the intervention group and 71 (86.6%) of the 82 participants in the control group. The primary outcome results, mean overall MANSA score, are reported in Table 2 and summarised in Supplementary Fig. 1. Mean change in overall MANSA score did not differ between the groups (intervention: 0.25, s.d. 0.73; control: 0.21, s.d. 0.86; estimated fully adjusted between-group difference 0.03, 95% CI −0.25 to 0.31; *P* = 0.819). There were no significant differences between the groups for the prespecified sensitivity analyses. The crude ICC for the primary outcome was 0.20 (95% CI 0.09–0.40) (see Supplementary Table 2 for crude, partially and fully adjusted ICCs for all outcomes). There was no statistically significant impact of the COVID-19 outbreak on the MANSA or TUS (Table 2 and Supplementary Table 3), nor was either the interaction effect between allocated group and COVID-19-affected categorisation (*P* = 0.225) or the COVID-19-affected categorisation covariate statistically significant (*P* = 0.225) (Supplementary Table 5). There was no evidence of differential treatment effects (i.e. no significant interaction effect between allocated group and subgroup) in any of the four planned subgroup analyses (region *P* = 0.205; practice size *P* = 0.791; screening location *P* = 0.136; participant diagnosis *P* = 0.386). Summary statistics of the MANSA separate domain ratings and general (single item) quality-of-life ratings are shown in Supplementary Table 5.

**Table 2:** Primary outcome, overall Manchester Short Assessment of Quality of Life score at baseline and follow-up: primary modified intention-to-treat analyses and prespecified sensitivity analyses

	Intervention (*n* = 116)			Control (*n* = 82)			Partially adjusted^a^ treatment effect^b^ (intervention – control) (95% CI) *P*-value	Fully adjusted^c^ treatment effect^b^ (intervention – control) (95% CI) *P*-value
	Baseline	Follow-up	Change	Baseline	Follow-up	Change		
Primary analysis: mITT	*n* = 116 4.29 (0.88) [2.18–6.36]	*n* = 99 4.54 (0.82) [2.45–6.80]	*n* = 99 0.25 (0.73) [−1.85 to 3.32]	*n* = 80 4.33 (0.99) [2.45–6.55]	*n* = 72 4.51 (1.01) [2.36–6.82]	*n* = 71 0.21 (0.86) [−2.45 to 2.40]	0.04 (−0.24 to 0.33) *P* = 0.762	0.03 (−0.25 to 0.31) *P* = 0.819
Sensitivity analysis: per protocol – follow-up window	*n* = 58 4.35 (0.88) [2.18–6.36]	*n* = 57 4.67 (0.83) [2.45–6.80]	*n* = 57 0.30 (0.76) [−1.85 to 3.32]	*n* = 35 4.20 (1.03) [2.45–6.18]	*n* = 36 4.43 (0.81) [2.91–5.82]	*n* = 35 0.26 (0.77) [−1.09 to 1.85]	0.10 (−0.23 to 0.43) *P* = 0.533	0.09 (−0.26 to 0.44) *P* = 0.602
Sensitivity analysis: per protocol – care partner availability	*n* = 98 4.27 (0.84) [2.40–6.36]	*n* = 85 4.47 (0.84) [2.45–6.80]	*n* = 85 0.23 (0.69) [−1.85 to 1.87]	*n* = 80 4.33 (0.99) [2.45–6.55]	*n* = 72 4.51 (1.01) [2.36–6.82]	*n* = 71 0.21 (0.86) [−2.45 to 2.40]	−0.004 (−0.30 to 0.29) *P* = 0.978	−0.01 (−0.32 to 0.30) *P* = 0.939
Sensitivity analysis: ITT - multiple imputation	–	–	–	–	–	–	0.06 (−0.22 to 0.34) *P* = 0.664	0.06 (−0.22 to 0.33) *P* = 0.691
Sensitivity analysis: pre COVID-19 participants only	*n* = 30 4.28 (0.98) [2.55–5.89]	*n* = 30 4.49 (0.78) [2.82–5.64]	*n* = 30 0.21 (0.69) [−1.09 to 1.87]	*n* = 22 4.24 (0.79) [2.64–5.90]	*n* = 22 4.15 (0.89) [2.36–5.82]	*n* = 22 −0.09 (0.74) [−1.20 to 1.45]	0.34 (−0.27 to 0.95) *P* = 0.245	0.17 (−0.51 to 0.84) *P* = 0.574
Sensitivity analysis: participants during COVID-19	*n* = 71 4.29 (0.87) [2.18–6.36]	*n* = 69 4.57 (0.84) [2.45–6.80]	*n* = 69 0.27 (0.76) [−1.85 to 3.32]	*n* = 49 4.35 (1.11) [2.45–6.55]	*n* = 50 4.67 (1.03) [2.50–6.82]	*n* = 49 0.34 (0.89) [−2.45 to 2.40]	−0.09 (−0.40 to 0.23) *P* = 0.570	−0.08 (−0.42 to 0.25) *P* = 0.611
CACE(i): minimum of six sessions of at least 10 min							0.05^d^ (−0.35 to 0.45) *P* = 0.795	0.02^d^ (−0.35 to 0.40) *P* = 0.910
CACE(ii): minimum of four sessions with goals discussed							0.05^d^ (−0.31 to 0.41) *P* = 0.796	0.02^d^ (−0.32 to 0.35) *P* = 0.910

Data are displayed as mean (s.d.) [range] unless otherwise specified. mITT, modified intention to treat; ITT, intention to treat; CACE, complier-average causal effect; MANSA, Manchester Short Assessment of Quality of Life.

a. Adjusted for baseline MANSA and including random effects for general practice.

b. Treatment effect is the between-group difference in the change in MANSA between baseline and follow-up.

c. Adjusted for baseline MANSA, stratification variables (practice size and locality) and including random effects for general practice.

d. The CACE estimate is the mean difference between those in the intervention group who complied with the intervention (CACE(i) *n* = 68 compliers; CACE (ii) *n* = 76 compliers), and those in the control group who would have complied had they been offered the intervention.

None of the secondary outcomes differed significantly between allocated groups (Table 3). There was no evidence of a statistically significant between-group difference in TUS (excluding two outliers) for participants who completed the study before the start of the COVID-19 pandemic compared with during the pandemic (Table 3). Additionally, neither the interaction effect between allocated group and COVID-19-affected categorisation (*P* = 0.211) or the COVID-19-affected categorisation covariate (*P* = 0.841) were statistically significant (Supplementary Table 4). Summary statistics for the additional measures captured are shown in Supplementary Table 5.

**Table 3:** Secondary outcomes at baseline and follow-up: primary modified intention-to-treat analyses and prespecified sensitivity analyses

	Intervention (*n* = 116)			Control (*n* = 82)			Partially adjusted^a^ treatment effect^b^ (intervention – control) (95% CI) *P*-value	Fully adjusted^c^ treatment effect^b^ (intervention – control) (95% CI) *P*-value	Crude ICC (95% CI)
	Baseline	Follow-up	Change	Baseline	Follow-up	Change			
TUS (hours)	*n* = 115 33.9 (24.3) [0.0–130.0]	*n* = 98 27.4 (24.3) [0.0–110.5]	*n* = 98 −8.3 (27.1) [−120.3 to 71.3]	*n* = 81⋅0 29.2 (25.5) [0.2–124.4]	*n* = 71 26.6 (22.7) [0.0–97.2]	*n* = 71 −2.5 (22.8) [−86.8 to 65.3]	−1.7 (−9.4 to 6.0) *P* = 0.661	−1.8 (−8.9 to 5.3) *P* = 0.612	0.007 (0–1)
TUS for pre COVID-19 participants only (hours)	*n* = 29 35.1 (18.7) [4.4–66.5]	*n* = 29 30.2 (19.2) [2.8–82.6]	*n* = 29 −4.9 (22.5) [−51.9 to 49.2]	*n* = 23 40.6 (34.8) [0.2–124.4]	*n* = 23 31.4 (25.7) [0.2–91.2]	*n* = 23 −9.2 (27.0) [−86.8 to 63.7]	2.2 (−11.8 to 16.2) *P* = 0.728	3.0 (−12.4 to 18.5) *P* = 0.651	–
TUS for COVID-19 affected participants only (hours)	*n* = 71 35.3 (26.5) [0.0–130.0]	*n* = 69 26.2 (26.2) [0.0–110.5]	*n* = 69 −9.8 (28.8) [−120.3 to 71.3]	*n* = 49 23.4 (18.3) [0.3–70.6]	*n* = 48 24.4 (21.0) [0.0–97.2]	*n* = 48 0.8 (20.0) [−46.6 to 65.3]	−3.8 (−14.2 to 6.7) *P* = 0.464	−5.6 (−15.1 to 4.0) *P* = 0.236	–
WEMWBS	*n* = 116 39.3 (12.0) [14–70]	*n* = 97 42.1 (10.7) [16–64]	*n* = 97 2.1 (8.4) [−26 to 37]	*n* = 80 39.8 (11.7) [14–70]	*n* = 71 41.5 (12.4) [14–66]	*n* = 70 2.6 (9.5) [−29 to 26]	−0.2 (−2.9 to 2.5) *P* = 0.868	−0.5 (−3.4 to 2.3) *P* = 0.713	0.12 (0.03–0.36)
SWEMWBS	*n* = 111 18.9 (4.4) [7.0–35.0]	*n* = 94 20.3 (4.3) [9.5–35.0]	*n* = 89 11 (3.7) [−11.3 to 17.8]	*n* = 76 19.1 (4.5) [7.0–35.0]	*n* = 69 19.9 (5.2) [7.0–30.7]	*n* = 65 1.0 (4.1) [−13.7 to 11.2]	0.2 (−1.1 to 1.4) *P* = 0.771	−0.01 (−1.3 to 1.3) *P* = 0.982	0.13 (0.04–0.37)
Brief INSPIRE	*n* = 61 Median: 50 IQR: [15–65]	*n* = 79 Median: 75 IQR: [55–90]	*n* = 43 Median: 30 [0–55]	*n* = 38 Median: 45 IQR: [15–55]	*n* = 22 Median: 47.5 IQR: [5–70]	*n* = 13 Median: 30 IQR: [−25 to 20]	Not applicable^d^	Not applicable^d^	Not applicable^d^
QPR-15	*n* = 112 29.3 (12.8) [0–60]	*n* = 95 34.8 (10.2) [9–56]	*n* = 91 4.6 (8.9) [−27 to 27]	*n* = 79 31.4 (12.4) [5.4–60]	*n* = 71 33.4 (12.7) [0–56]	*n* = 70 2.5 (10.0 ) [−21 to 30.6]	1.8 (−1.0 to 4.6) *P* = 0.194	1.5 (−1.3 to 4.3) *P* = 0.269	0.11 (0.03–0.37)
ICECAP-A	*n* = 112 0.61 (0.22) [0.00–1.00]	*n* = 98 0.67 (0.21) [0.08–0.97]	*n* = 94 0.05 (0.15) [−0.36 to 0.48]	*n* = 77 0.63 (0.22) [0.00–1.00]	*n* = 71 0.64 (0.23) [0.15–0.97]	*n* = 67 0.02 (0.18) [−0.48 to 0.49]	0.03 (−0.02 to 0.08) *P* = 0.248	0.02 (−0.03, 0.08) *P* = 0.346	0.07 (0.008–0.38)
EQ-5D-5L	*n* = 111 0.51 (0.33) [−0.25 to 1.00]	*n* = 98 0.54 (0.31) [−0.20 to 1.00]	*n* = 93 0.04 (0.22) [−0.44 to 0.68]	*n* = 77 0.52 (0.31) [−0.25 to 1.00]	*n* = 72 0.51 (0.28) [−0.22 to 0.88]	*n* = 68 −0.01 (0.31) [−0.81 to 0.89]	0.04 (−0.04 to 0.12) *P* = 0.267	0.05 (−0.03 to 0.13) *P* = 0⋅243	0.08 (0.01–0.36)

Data are displayed as mean (s.d.) [range], unless otherwise specified. ICC, intracluster correlation; TUS, Time Use Survey; WEMWBS, Warwick-Edinburgh Mental Wellbeing Scale; SWEMWBS, Short Warwick-Edinburgh Mental Wellbeing Scale; Brief INSPIRE, XXX; QPR-15, Questionnaire about the Process of Recovery; ICECAP-A, ICEpop CAPability measure; EQ-5D-5L, five-level EuroQol five-dimensional.

a. Adjusted for baseline MANSA and including random effects for general practice.

b. Treatment effect is the between-group difference in the change in MANSA between baseline and follow-up.

c. Adjusted for baseline MANSA, stratification variables (practice size and locality) and including random effects for general practice.

d. Planned inferential analyses not undertaken because of high levels of missing data for the Brief INSPIRE outcome measure.

Safety outcomes and serious adverse events are summarised in Supplementary Table 6. There were seven acute mental health episodes recorded (three classified as ‘treated’ and four as ‘untreated’, according to protocol criteria). Twenty-eight serious adverse events in 18 participants were reported. Eleven serious adverse events were categorised as related to psychiatric disorders. None of the serious adverse events were deemed to be related to the intervention or trial procedures.

## Discussion

The PARTNERS2 study is the first definitive, randomised controlled trial of a coaching-enhanced collaborative care intervention for individuals with SMI. A majority of recruited participants had significant needs and were not receiving expert mental health support. The PARTNERS intervention was established in low-income urban, coastal and rural communities, and maintained in pandemic conditions. Although 91% of participants in the intervention group received at least one contact with a care partner and 87% had goals assessed, there were five practices where care partners were present for <70% of the intervention period. Outcome measures were analysed for the 174 participants (88%) with follow-up data, much of which were collected by remote means because of the COVID-19 pandemic. No differences were found between control and intervention groups for the primary outcome measure (MANSA), the secondary outcomes or the planned sensitivity analyses. There were no observable differences between groups for key safety data.

The strengths of the trial included its pragmatic real-life design, with high proportions of individuals from areas of deprivation. We achieved high levels of follow-up and no reduction in contact rate during the pandemic. Although the number of participants recruited was less than originally planned, the revised recruitment target, despite being calculated for 80% power, gave data from a sufficient number of participants to enable between-group differences to be estimated with high levels of precision. The PARTNERS intervention was informed by existing literature, expert interviews, input from patients and the public, and a formative evaluation during the feasibility phase.

There are limitations in trial design and delivery. The cluster randomised controlled trial design resulted in an uneven allocation of individuals. There was more variation in practice cluster size than anticipated, and although practice size was used as a minimisation factor, it was less correlated with the number of individuals recruited than expected. There were significant recruitment challenges at the start of the trial, with enhancements made to recruitment protocols to allow practices to engage more easily in the research and to increase the opportunities for individuals who might otherwise not have been reached. The high proportion of participants with a diagnosis of bipolar disorder rather than schizophrenia in this sample contrasts with proportions of those in mental health services;^29^ it could represent higher rates of discharge to primary care for this group, or that it is easier and/or more acceptable to engage those with bipolar disorder in research. We also recruited fewer participants from Black and minority ethnic communities than expected,^29^ which further reduced real-world applicability of findings. The high proportion of intervention participants being ‘under primary care only’ could have led to a differential treatment effect, but this was explored in prespecified subgroup analyses. Blinding was not possible for researchers or participants. The delivery of the intervention was affected by pressures within the NHS, including illness, delays replacing care partners and supervisors, and supervisors not being able to prioritise supervision practice. At times, the research team had to step in to provide supervision. However, this reflects the current reality of the NHS.

Outcome selection was based on an analysis of the intervention theory and predicted stages of change. However, selection of a primary outcome was not straightforward because of the wide range of different outcomes the intervention was designed to have the potential to change. Although mean baseline scores of outcomes indicate significant need, the absence of a maximum MANSA score for inclusion in the study meant that some individuals had relatively positive MANSA scores at baseline, potentially making improvements more difficult to detect in the primary outcome. Additionally, there was difficulty collecting two of the planned outcome measures. The Brief INSPIRE measure, aiming to examine experience of recovery orientated care, was not formally analysed because of substantial between-site differences in rates of missing data. Key physical healthcare monitoring data were unable to be collected from health records because of reductions in researcher presence in practices during pandemic conditions. Notably, with person-centred interventions, goals and changes are individual and idiosyncratic and, however important to the individual, may not be picked up by standard outcome measures. Alternatively, it could be argued that asking care partners – who provide relatively low-intensity care, albeit with the support of a wider team – to address physical, social and psychological needs that may have accrued over years is too great a challenge. This is, however, an aim of care coordination and community mental health team care, and a key outcome of the Community Mental Health Framework policy.^10^

Difficulties with intervention delivery, imbalance in numbers of individual participants randomised, a heterogeneous group of participants and potential lack of sensitivity of standard outcome measures to the often unpredictable path to recovery makes full interpretation complex. However, a shift from usual to PARTNERS care in England in the period 2017–2020 did not result in a clinically significant improvement in standard outcome measures. Although the study was not designed as a non-inferiority trial, the lack of difference in outcomes, measured to a high degree of precision, and lack of significant imbalance in safety data suggest that the shift to PARTNERS care is not likely to be harmful. This is important, given the current policy direction of more integrated, primary care-based, person-centred care across the UK and in many other countries globally.

There are few other comparable studies. There are no published trials of coaching alone for psychosis, rather than as part of collaborative care, and studies of motivational practices tend to focus on medication adherence. Collaborative care for individuals with depression and anxiety is associated with a modest but significant improvement in outcomes compared with usual care.^30^ Collaborative care for those with depression and physical comorbidity has a small average effect size. In our updated Cochrane review,^12^ interventions varied, most did not meet a strict definition of collaborative care and the majority of evidence was either low or very low quality, with no overall conclusions about effectiveness. Our neutral result is broadly in keeping with these results.

In conclusion, this was the first definitive randomised controlled trial of a coaching-based collaborative care intervention for people with SMI. It was completed in a challenging health system and pandemic context. No improvements in standard outcome measures were seen, but the shift to primary care-based care did not lead to increased mental health crises. The qualitative process evaluation analyses will cast more light on how best to target and support implementation of coaching-based collaborative care. For system leaders redesigning services, the message is that the PARTNERS collaborative care model (including for those currently receiving low-intensity secondary care) is likely to be safe, but in its current intensity, is unlikely to generate major improvements in quality of life within a year.

## Data Availability

Anonymised data may be made available by request to corresponding author. The study protocol has been published and a link to statistical analysis plan is included in the paper. CONSORT 2010 guidelines were followed in the reporting of this trial. Several authors had full access to all the data in the study and had final responsibility for the decision to submit for publication. Changes from the original funding proposal include, following pilot work, a funded extension for a full trial (rather than an external pilot trial as originally funded), which is described in the published protocol, trial registry and statistical analysis plan. The discrepancies from the published protocol included provision of top-up training for existing practitioners during the trial and changes made in response to COVID-19, including online delivery and remote data collection. The study protocol and statistical analysis plan have been published.

## Supplementary material

For supplementary material accompanying this paper visit https://doi.org/10.1192/bjp.2023.28. click here to view supplementary material
