# Supplementary material for: The effectiveness of a primary care-based collaborative care model to improve quality of life in people with severe mental illness: PARTNERS2 cluster randomised controlled trial
Source: Br J Psychiatry. 2023 Jun;222(6):246–56. doi: 10.1192/bjp.2023.28 (PMC10201334; doi:10.1192/bjp.2023.28)
Supplement: Supplementary file 1 [file S0007125023000284sup001.doc]

# Supplementary Material

# Contents Page

Supplementary figure S1. Summary of primary and sensitivity analyses of primary outcome….…………………2

Supplementary table S1. Participants’ interactions with Care Partner 3

Supplementary table S2. Estimates of Intraclass Correlation Coefficients (ICC) for primary and secondary outcomes ……………………………………………………………………………………………………..…...3

Supplementary table S3. Additional COVID-19 Sensitivity Analysis of MANSA and TUS ….. 4

Supplementary table S4. Summary statistics of additional components of MANSA 5

Supplementary table S5. Summary statistics of the additional collected measures (not subject to inferential analysis) 8

Supplementary table S6. Safety Outcomes and Serious Adverse Events 9

**Figure S1 – Summary of primary and sensitivity analyses of primary outcome**


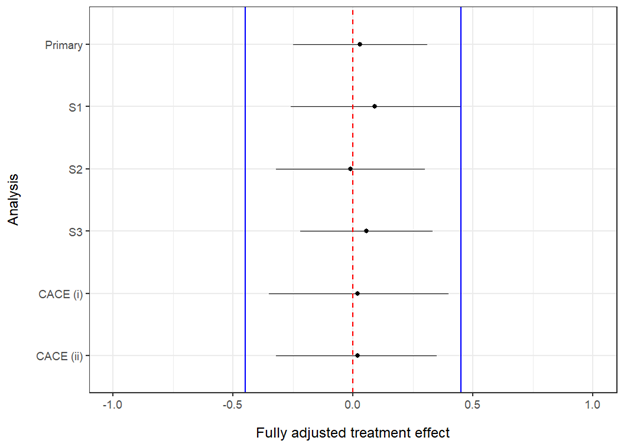


Fully adjusted between-group difference in the change in MANSA between baseline and follow-up for the primary analysis, per protocol and multiple imputation sensitivity analyses, and Complier Average Causal Effect (CACE) analyses.

The treatment effect is adjusted for baseline MANSA, stratification variables (practice size and locality) and including random effects for GP practice. Error bars represent 95% confidence intervals. Blue vertical lines represent the target difference in MANSA score (0·45).

S1 = per protocol sensitivity analysis of follow-up window; S2 = per protocol sensitivity analysis of care partner availability; S3 = multiple imputation.

The CACE estimate is the mean difference between those in the intervention group who complied with the intervention, and those in the control group who would have complied had they been offered the intervention. CACE (i) = minimum of six sessions of at least ten minutes; CACE (ii) = minimum of four sessions with goals discussed.

# Supplementary table S1. Participants’ interactions with Care Partner

| Type of care-partner interaction | **N (%) participants with at least one interaction with the Care Partner** | **Median (interquartile range) [min, max] of the number of interactions with the Care Partner** |
| --- | --- | --- |
| All modes | 106/116 (91·4%) | 12 (7·3, 19·8) [1, 40] |
| Telephone or face-to-face (including virtual) lasting at least 10 minutes | 103/116 (88·8%) | 9 (5, 12·5)  [1, 25] |

**Supplementary table S2: Estimates of Intraclass Correlation Coefficients (ICC) for primary and secondary outcomes**

| **Outcome** | **Crude ICC^1^ (95% CI)** | **Partially Adjusted^2^ ICC (95% CI)** | **Fully Adjusted^3^ ICC (95% CI)** |
| --- | --- | --- | --- |
| MANSA | 0·20 (0·09, 0·40) | 0·14 (0·04, 0·36) | 0·13 (0·04, 0·36) |
| WEMWBS | 0·12 (0·03, 0·36) | 0·002 (0, 1) | 0·02 (0·00005, 0·96) |
| SWEMWBS | 0·13 (0·04, 0·37) | 0 | 0 |
| QPR-15 | 0·11 (0·03, 0·37) | 0 | 0 |
| TUS | 0·007 (0, 1) | 0·06 (0·007, 0·35) | 0·01 (0, 0·98) |
| ICECAP-A | 0·07 (0·008, 0·38) | 0 | 0 |
| EQ-5D-5L | 0·08 (0·01, 0·36) | 0·0007 (0,1) | 0·003 (0,1) |

1 Modelling the outcome score at follow-up, with no adjustments, and including random effects for GP practice 2 Modelling the change, and adjusted for baseline and including random effects for GP practice

3 Modelling the change, and adjusted for baseline as well as the stratification variables (practice size and locality) and including random effects for GP practice

# Supplementary table S3. Additional COVID-19 Sensitivity Analysis of MANSA and TUS

| N; Mean (SD) [Range] |  | Intervention (N =101^3^) |  |  | Control (N = 73^3^) |  | Partially Adjusted^4^ Treatment Effect^5^ (Intervention – Control) (95% CI)  p-value | Partially Adjusted^4^ COVID EFFECT (95% CI)  p-value | Fully Adjusted^6^ Treatment Effect^5^ (Intervention – Control) (95% CI)  p-value | Fully Adjusted^6^ COVID EFFECT  ** (95% CI)  p-value |
| --- | --- | --- | --- | --- | --- | --- | --- | --- | --- | --- |
|  | Baseline | Month 10 | Change | Baseline | Month 10 | Change |  |  |  |  |
| ***MANSA*** | | | | | | | | | | |
| Pre COVID-19  participants only^1^ | N = 30 4·28 (0·98) [2·55, 5·89] | N = 30 4·49 (0·78) [2·82, 5·64] | N = 30 0·21 (0·69) [-1·09, 1·87] | N = 22 4·24 (0·79) [2·64, 5·90] | N = 22 4·15 (0·89) [2·36, 5·82] | N = 22  -0·09 (0·74) [-1·20, 1·45] | 0·34  (-0·27, 0·95) p = 0·245 | -0·42*  (-0·99, 0·16) p =0·150 | 0·17  (-0·51, 0·84) p = 0·574 | -0·36*  (-0·95, 0·23) p = 0·225 |
| COVID-19  affected participants only^2^ | N = 71 4·29 (0·87)  [2·18, 6·36] | N = 69 4·57 (0·84)  [2·45, 6·80] | N = 69 0·27 (0·76)  [-1·85, 3·32] | N = 49 4·35 (1·11)  [2·45, 6·55] | N = 50 4·67 (1·03)  [2·50, 6·82] | N = 49 0·34 (0·89)  [-2·45, 2·40] | -0·09  (-0·40, 0·23) p = 0·570 |  | -0·08  (-0·42, 0·25) p = 0·611 |  |
| All Participants | N = 116 4·29 (0·88)  [2·18, 6·36] | N = 99 4·54 (0·82)  [2·45, 6·80] | N = 99 0·25 (0·73)  [-1·85, 3·32] | N = 80 4·33 (0·99)  [2·45, 6·55] | N = 72 4·51 (1·01)  [2·36, 6·82] | N = 71 0·21 (0·86)  [-2·45, 2·40] | 0·04  (-0·24, 0·32) p = 0·766 | 0·23**  (-0·06, 0·52) p = 0·114 | 0·03  (-0·25, 0·31) p = 0·823 | 0·18**  (-0·12, 0·49) p = 0·225 |
| ***Time Use Survey*** | | | | | | | | | | |
| Pre COVID-19^1^ | N = 29 35·1 (18·7)  [4·4, 66·5] | N = 29 30·2 (19·2)  [2·8, 82·6] | N = 29  -4·9 (22·5) [-51·9, 49·2] | N = 23 40·6 (34·8)  [0·2, 124·4] | N = 23 31·4 (25·7)  [0·2, 91·2] | N = 23  -9·2 (27·0) [-86·8, 63·7] | 2·2  (-11·8, 16·2) p = 0·728 | -6·7  (-23·4, 9·9) p = 0·418 | 3·0  (-12·4, 18·5)  p = 0·651 | -9·9  (-25·7, 5·9) p = 0·211 |
| During COVID- 19^2^ | N = 71  35·3 (26·5) [0·0, 130·0] | N = 69  26·2 (26·2) [0·0, 110·5] | N = 69  -9·8 (28·8) [-120·3,71·3] | N = 49  23·4 (18·3) [0·3, 70·6] | N = 48  24·4 (21·0) [0·0, 97·2] | N = 48 0·8 (20·0)  [-46·6, 65·3] | -3·8  (-14·2, 6·7) p = 0·464 |  | -5·6  (-15·1, 4·0) p = 0·236 |  |
| All Participants | N = 115 33·9 (24·3)  [0·0, 130·0] | N = 98 27·4 (24·3)  [0·0, 110·5] | N = 98  -8·3 (27·1)  [-120·3, 71·3] | N = 81 29·2 (25·5)  [0·2, 124·4] | N = 71 26·6 (22·7)  [0·0, 97·2] | N = 71  -2·5 (22·8) [-86·8, 65·3] | -1·5  (-9·3, 6·2) 0·687 | -2·8  (-11·0, 5·4) p = 0·492 | -1·7  (-9·0, 5·6) p = 0·632 | -0·8  (-8·8, 7·2) p = 0·841 |

1. Includes participants providing follow-up data before lockdown restrictions commenced in the UK on 23 March 2020
2. Includes participants providing follow-up data on or after lockdown restrictions commenced in the UK on 23 March 2020 3 The number of participants who attended both baseline and follow-up
3. Adjusted for baseline and including random effects for GP practice
4. Treatment effect is the between-group difference in the change in outcome between baseline and primary endpoint where available 6 Adjusted for baseline as well as the stratification variables (practice size and locality) and including random effects for GP practice

***** Allocation X COVID-19 status interaction term; ** COVID-19 affected categorisation covariate coefficient

# Supplementary table S4: Summary statistics of additional components of MANSA

|  |  | Intervention (N = 116) |  |  | Control (N = 82) |  |
| --- | --- | --- | --- | --- | --- | --- |
|  | Baseline | Follow-up | Change | Baseline | Follow-up | Change |
| Life as a whole, today | n = 114 3·8 (1·6) [1, 7] | n = 98 4·2 (1·6) [1, 7] | n = 96 0·26 (1·4) [-4, 4] | n = 78 4·1 (1·7) [1, 7] | n = 72 4·2 (1·6) [1, 7] | n = 68 0·26 (1·7) [-4, 6] |
| Life as a whole | n = 116 3·7 (1·6) [1, 7] | n = 97 4·3 (1·5) [1, 7] | n = 97 0·40 (1·4) [-3, 4] | n = 80 3·9 (1·5) [1, 7] | n = 72 4·1 (1·7) [1, 7] | n = 71 0·24 (1·5) [-4, 5] |
| **N (%) Restricted life opportunities** |  |  |  |  |  |  |
| - *Work/Education* | 53/116 (45·7%) | 40/100 (40·0%) |  | 41/82 (50·0%) | 31/73 (42·5%) |  |
| - *Finances* | 65/116 (56·0%) | 47/100 (47·0%) |  | 41/82 (50·0%) | 36/73 (49·3%) |  |
| - *Leisure* | 48/116 (41·4%) | 42/100 (42·0%) |  | 36/82 (43·9%) | 34/73 (46·6%) |  |
| - *Social Life* | 66/116 (56·9%) | 51/100 (51·0%) |  | 40/82 (48·8%) | 32/73 (43·8%) |  |
| - *Living Situation* | 46/116 (39·7%) | 32/100 (32·0%) |  | 30/82 (36·6%) | 24/73 (32·9%) |  |
| - *Family Life* | 50/116 (43·1%) | 33/100 (33·0%) |  | 32/82 (39·0%) | 26/73 (35·6%) |  |
| - *Safety* | 19/116 (16·4%) | 19/100 (19·0%) |  | 23/82 (28·0%) | 20/73 (2·.4%) |  |
| - *Health* | 81/116 (69·8%) | 64/100 (64·0%) |  | 56/82 (68·3%) | 54/73 (74·0%) |  |
| Months in work in the past two years | n = 96  5·9 (9·4) [0, 24] | n = 85  6·1 (9·8) [0, 24] | n = 70  0·61 (3·7) [12, 14] | n = 62  5·1 (9·2) [0, 24] | n = 68  3·9 (8·2) [0, 24] | n = 49  -0·98 (3·4) [-15, 3] |
| Average hours working per week | n = 34 23·6 (17·1)  [0, 56] | n = 23 27·6 (11·8)  [2, 40] | n = 18  -0·81 (6·2) [-12, 14] | n = 25 18·3 (15·7)  [0, 38] | n = 17 25·0 (15·5)  [0, 40] | n = 14 3·9 (11·4)  [-8, 37] |
| **N (%) Employment Status** |  |  |  |  |  |  |
| - *Paid Work* | 26/104 (25·0%) | 22/92 (23·9%) |  | 16/65 (24·6%) | 14/68 (20·6%) |  |
| - *Training/education* | 0/104 (0·0%) | 3/92 (3·3%) |  | 3/65 (4·6%) | 4/68 (5·9%) |  |
| - *Not working – illness* | 36/104 (34·6%) | 36/92 (39·1%) |  | 25/65 (38·5%) | 31/68 (45·6%) |  |
| - *Not working – looking for work* | 6/104 (5·8%) | 4/92 (4·3%) |  | 2/65 (3·1%) | 1/68 (1·5%) |  |
| - *Unemployed* | 12/104 (11·5%) | 9/92 (9·8%) |  | 8/65 (12·3%) | 6/68 (8·8%) |  |
| - *Retired* | 19/104 (18·3%) | 18/92 (19·6%) |  | 6/65 (9·2%) | 9/68 (13·2%) |  |

| - *Other* | 5/104 (4·8%) | 0/92 (0·0%) | 5/65 (7·7%) | 3/68 (4·4%) |
| --- | --- | --- | --- | --- |
| **N (%) difficulty meeting household bills** |  |  |  |  |
| - *All of the time* | 20/110 (18·2%) | 8/96 (8·3%) | 13/80 (16·3%) | 10/72 (13·9%) |
| - *Most of the time* | 15/110 (13·6%) | 12/96 (12·5%) | 11/80 (13·8%) | 10/72 (13·9%) |
| - *Some of the time* | 30/110 (27·3%) | 19/96 (19·8%) | 18/80 (22·5%) | 17/72 (23·6%) |
| - *Seldom* | 19/110 (17·3%) | 25/96 (26·0%) | 19/80 (23·8%) | 12/72 (16·7%) |
| - *Never* | 26/110 (23·6%) | 32/96 (33·3%) | 19/80 (23·8%) | 23/72 (31·9%) |
| N (%) have a close friend | 86/116 (74·1%) | 70/97 (72·2%) | 58/80 (72·5%) | 55/73 (75·3%) |
| N (%) seen a friend in the past week | 73/115 (63·5%) | 49/97 (50·5%) | 46/77 (59·7%) | 40/73 (54·8%) |
| **N (%) frequency of contact with a relative** |  |  |  |  |
| - *Not at all* | 8/114 (7·0%) | 6/96 (6·3%) | 6/80 (7·5%) | 4/72 (5·6%) |
| - *Daily* | 39/114 (34·2%) | 41/96 (42·7%) | 30/80 (37·5%) | 21/72 (29·2%) |
| - *At least weekly* | 46/114 (40·4%) | 32/96 (33·3%) | 32/80 (40·0%) | 36/72 (50·0%) |
| - *At least monthly* | 6/114 (5·3%) | 10/96 (10·4%) | 4/80 (5·0%) | 7/72 (9·7%) |
| - *At least 3 monthly* | 11/114 (9·6%) | 4/96 (4·2%) | 1/80 (1·3%) | 0 (0·0%) |
| - *At least yearly* | 3/114 (2·6%) | 2/96 (2·1%) | 3/80 (3·8%) | 2/72 (2·8%) |
| - *Less than yearly* | 1/114 (0·9%) | 1/96 (1·0%) | 4/80 (5·0%) | 2/72 (2·8%) |
| **N (%) Living Situation** |  |  |  |  |
| - *House/flat (owned)* | 53/116 (45·7%) | 46/98 (46·9%) | 23/80 (28·8%) | 23/71 (32·4%) |
| - *House/flat (rented)* | 55/116 (47·4%) | 46/98 (46·9%) | 52/80 (65·0%) | 42/71 (59·2%) |
| - *Mobile home* | 1/116 (0·9%) | 2/98 (2·0%) | 0 (0·0%) | 0 (0·0%) |
| - *Hostel/supported/group home* | 3/116 (2·6%) | 1/98 (1·0%) | 1/80 (1·3%) | 0 (0·0%) |
| - *Sheltered housing* | 1/116 (0·9%) | 3/98 (3·1%) | 3/80 (3·8%) | 6/71 (8·5%) |
| - *Residential home* | 2/116 (1·7%) | 0 (0·0%) | 0 (0·0%) | 0 (0·0%) |
| - *Homeless* | 1/116 (0·9%) | 0 (0·0%) | 1/80 (1·25%) | 0 (0·0%) |
| **N (%) Cohabitees** |  |  |  |  |
| - *Alone* | 49/116 (42·2%) | 40/100 (40·0%) | 40/82 (48·8%) | 38/73 (52·1%) |
| - *Spouse/partner* | 45/116 (38·8%) | 39/100 (39·0%) | 23/82 (28·0%) | 22/73 (30·1%) |
| - *Parent(s)* | 8/116 (6·9%) | 8/100 (8·0%) | 3/82 (3·7%) | 1/73 (1·4%) |
| - *Children under 18* | 20/116 (17.2%) | 20/100 (20.0%) | 11/82 (13.4%) | 10/73 (13.7%) |
| - *Children over 18* | 9/116 (7·8%) | 7/100 (7·0%) | 12/82 (14·6%) | 7/73 (9·6%) |

| - *Other family* | 6/116 (5·2%) | 5/100 (5·0%) | 3/82 (3·7%) | 3/73 (4·1%) |
| --- | --- | --- | --- | --- |
| - *Non-family* | 1/116 (0·9%) | 0 (0·0%) | 3/82 (3·7%) | 4/73 (5·5%) |
| N (%) Victim of violence in the past year | 8/115 (7·0%) | 7/96 (7·3%) | 6/76 (7·9%) | 7/72 (9·7%) |

Data are n/N (%) or mean (SD) and [range].

**Supplementary table S5: Summary statistics of the additional collected measures (not subject to inferential analysis)**

|  |  | **Intervention (N = 103)** |  |  | **Control (N = 73)** |  |
| --- | --- | --- | --- | --- | --- | --- |
|  | **Baseline** | **Follow-up** | **Change** | **Baseline** | **Follow-up** | **Change** |
|  |  |  | ***Lifestyle Outcomes*** |  |  |  |
| **N (%) Smoker** | 51/116 (44%) | 39/100 (39%) | - | 31/82 (38%) | 27/72 (38%) | - |
| **Number of times smoking** | N = 48 | N = 39 | N = 35 | N = 28 | N = 27 | N = 22 |
| **per day** | 17·7 (12·3) | 17·5 (10·2) | -1·2 (7·3) | 17·1 (12·1) | 19·6 (13·2) | -1·5 (10·1) |
|  | [1, 60] | [2, 45] | [-22, 15] | [2, 60] | [2, 72] | [40, 16] |
| **N (%) Drinker** | 55/116 (47%) | 40/100 (40%) | - | 36/82 (44%) | 34/72 (47%) | - |
| **Number of times drinking** | N = 45 | N = 38 | N = 30 | N = 30 | N = 33 | N = 22 |
| **per week** | 2·9 (2·2) | 2·4 (2·2) | 0·08 (1·2) | 2·7 (2·3) | 2·6 (2·1) | -0·2 (1·7) |
|  | [0, 7] | [0, 7] | [-2, 4] | [0,7] | [0, 7] | [-5, 4] |
| **N (%) Cannabis user** | 7/116 (6%) | 6/99 (6%) | - | 7/82 (9%) | 6/72 (8%) | - |

# Supplementary table S6. Safety Outcomes and Serious Adverse Events

|  | Treated^1^ | Not Treated^2^ | Unknown^3^ |
| --- | --- | --- | --- |
| **Safety Outcomes** | | |  |
| Number of episodes | 3 | 4 | 0 |
| Number of episodes with inpatient admission | 2 | 2 | 0 |
| Number of episodes requiring home based crisis care | 3 | 4 | 0 |
| **Serious Adverse Events (SAEs)** | | |  |
| Number of SAEs | 13 | 13 | 2 |
| SAE organ system |  |  |  |
| - *Psychiatric disorder* | 4 | 7 | 0 |
| - *Other* | 9 | 6 | 2 |
| SAE Classification |  |  |  |
| - *Required hospitalisation* | 9 | 6 | 2 |
| - *Significant medical event* | 4 | 4 | 0 |
| - *Hospitalisation & significant medical event* | 0 | 2 | 0 |
| - *Death* | 0 | 1 | 0 |
| SAE Severity |  |  |  |
| - *Mild* | 1 | 0 | 0 |
| - *Moderate* | 10 | 8 | 1 |
| - *Severe* | 2 | 5 | 1 |
| SAE Relatedness |  |  |  |
| - *Unlikely* | 2 | 6 | 1 |
| - *Not related* | 11 | 7 | 1 |

^1^ An event is classified as “Treated” if it was experienced by a participant in the intervention arm *after* their first recorded interaction with a care partner

^2^ An event is classified as “Not Treated” if: (i) it was experienced by a participant allocated to the usual care arm or (ii) if it was experienced by a participant in the intervention arm *before* their first recorded interaction with a care partner.

^3^ An event was classified as “Unknown” if it was experienced by a participant allocated to the intervention arm, but there was no recorded date of onset of the event.
